# Repeated thyroid function evaluations in the dog: a retrospective study of 8,309 dogs

**DOI:** 10.3389/fvets.2025.1653398

**Published:** 2025-09-15

**Authors:** Anita M. Oberbauer, Janelle M. Belanger, Brian K. Petroff, Diane E. Brown, Christopher R. Wolfe, Thomas R. Famula

**Affiliations:** ^1^Department of Animal Science, University of California Davis, Davis, CA, United States; ^2^Michigan State University Veterinary Diagnostic Laboratory, Department of Pathobiology and Diagnostic Investigation, Michigan State University, East Lansing, MI, United States; ^3^Labcorp Early Development Laboratories, Inc., Madison, WI, United States; ^4^Orthopedic Foundation for Animals, Columbia, MO, United States

**Keywords:** canine, dog, thyroid, lymphocytic thyroiditis, breed

## Abstract

Hypothyroidism in dogs is a common diagnosis with some breeds being more prone to the condition. Autoimmune thyroiditis has an inherited component. Breeders wish to reduce the incidence by any means possible. Currently, the only opportunity lies in phenotypic testing of thyroid functionality. This retrospective study evaluated thyroid hormone and thyroglobulin autoantibodies (TgAA) analyses in dogs assessed multiple times to determine if the outcome changed over time. Data were extracted from the Orthopedic Foundation for Animals (OFA) database for 8,309 dogs which had been evaluated two or more times and the initial classification was compared to a final classification. More than 90% of dogs evaluated as normal for thyroid function remained normal in follow-up assessments. The greatest change was seen for dogs initially evaluated as equivocal; this was followed by a compensative autoimmune thyroiditis diagnosis being revised to normal, though 50% of the latter evaluation remained classified as compensative autoimmune thyroiditis. This suggests the presence of low levels of autoantibodies may be transient and that a dog presenting with autoantibodies should be reevaluated to confirm the development of autoimmune thyroiditis.

## Introduction

Thyroid hormones are vitally important to normal metabolism and physiological function including the regulation of growth and development. Yet, hypothyroidism in the dog is a common diagnosis (1–3) and some suggest that hypothyroidism may be over-diagnosed (3, 4). The vague and gradual onset of clinical signs associated with a reduction or lack of thyroid hormone, such as changes to metabolism with associated weight gain, lethargy, and hair loss (5), can result in clinical measurement of circulating thyroid hormones. Reliance on a subset of circulating thyroid hormones, which may be influenced by many factors including physiological status, diet, medications, age, and even breed of dog (6–12) is insufficient to truly diagnose hypothyroidism and may factor into an over-diagnosis of the condition. A comprehensive veterinary assessment of the dog including measures of circulating free and bound thyroid hormones as well as thyroid stimulating hormone are necessary to accurately diagnose hypothyroidism.

Most observed thyroid deficits are associated with primary hypothyroidism which can be caused by either infiltration of cells associated with an immune response, such as lymphocytes and macrophages, and are therefore referred to as lymphocytic thyroiditis, or by the replacement of the thyroid parenchyma with adipose and connective tissue which is referred to as idiopathic hypothyroidism. In the latter situation, the cause for the replacement is unknown although most hypothesize that idiopathic hypothyroidism is the final consequence of lymphocytic thyroiditis (4, 5, 13). Lymphocytic thyroiditis, or autoimmune thyroiditis, can be inherited (14, 15) with particular breeds more susceptible (2, 16). As its name implies, autoimmune thyroiditis is a consequence of thyroglobulin autoantibodies (TgAA) attacking the thyroid gland, resulting in its gradual destruction and inability to synthesize necessary thyroid hormones (17). Attempts to identify causal genetic contributors have had limited success (18), leaving phenotypic testing as the most reliable option for screening.

The development of lymphocytic thyroiditis (autoimmune thyroiditis) is characterized by a progression through stages that reflect the initial presence of TgAA, gradual decline in thyroid function with a reduction in circulating free T4 (thyroxine) secretion, a rise in thyroid stimulating hormone (TSH) in an effort to restore T4 levels, and a final stage wherein the thyroid tissue is fully atrophied, TgAAs are absent, T4 levels are very low, and TSH may be elevated or within normal reference values (1, 3, 5, 14, 16). The initial diagnostic, the presence of TgAAs, precedes clinical signs and changes in circulating thyroid hormones (16, 17). The duration between the presence of TgAAs and clinical signs is variable ranging from 12 to 18 months in dogs and sufficient diagnostic TgAAs are present by 24 months of age (14). This has led to the recommendation of thyroid testing for TgAAs at a minimum of 12 months of age with periodic retesting^1^ and testing prior to 12 months of age is typically uninformative because very few dogs have TgAAs at that age (14).

Appropriate phenotypic testing using a full thyroid profile for canine breeding stock at the suggested screening age may reduce the incidence of hypothyroidism (16). While a genetic test would be preferred, at this time no definitive causal variants have been identified in the development of canine autoimmune thyroiditis. Previous work has shown an association between immune genes, specifically dog leukocyte antigen (DLA) class II alleles, and hypothyroidism (18, 19) although the variants in DLA class II genes alone are insufficient to account for genetic susceptibility. Another study found a shared risk haplotype for hypothyroidism across several breeds that was not associated with the DLA region (20) while a whole-genome sequencing study in Giant Schnauzers revealed a deletion between two interferon alpha genes that is strongly associated with protection against hypothyroidism (15). Like many complex diseases, the causative mutation for canine hypothyroidism is yet to be discovered, leaving phenotypic testing as the sole approach to reducing incidence.

At present, the most effective screening tool for canine hypothyroidism is assessing for the presence of TgAAs. Yet some of the screening results are ambiguous or indicate subclinical thyroiditis based on the presence of autoantibodies but normal/euthyroid hormone levels. One study followed a small cohort of dogs initially diagnosed as subclinical and found that approximately one-third of the dogs remained subclinical with normal reference ranges for thyroid hormones, one-third were on thyroid hormone replacement therapy, and the remainder were either considered normal for thyroid function or still ambiguous (equivocal) (21). The present longitudinal retrospective study sought to characterize changes in thyroid parameters in a large cohort of dogs evaluated multiple times to understand better the implications of phenotypic screening for use in breeding decisions and canine health. We hypothesize that serial testing allows for more accurate detection of hypothyroidism in dogs. Additionally, we hypothesize that serial testing may lead to a high rate of reclassification in dogs having ambiguous initial testing results.

## Methods

The complete Orthopedic Foundation for Animals (OFA) thyroid database was queried for dogs, either pedigree or mixed breed, having more than one thyroid evaluation. Data evaluated included those publicly released and those withheld from public release from April 1996 through July 2023. Only test results from approved and certified testing laboratories are accepted by OFA. The certification criteria for meeting the requirements for OFA include “quality control, quality assurance and reagent certification…A site visit by a qualified veterinary endocrinologist chosen by the OFA will be required and continued quality assurance and quality control will be necessary to maintain certification. Fully certified status can be obtained by passing the site visit and passing the results of the first OFA quality assurance assay result test” (https://ofa.org/diseases/thyroid/thyroid-labs/ accessed June 16, 2025). The OFA categorizes thyroid results as normal, equivocal, positive compensative autoimmune thyroiditis (sometimes referred to as subclinical thyroiditis), idiopathically reduced thyroid function (hereafter referred to as idiopathic hypothyroidism in this manuscript reflective of the OFA reports), and positive autoimmune thyroiditis based upon criteria established in published literature (16, 22). The classifications reflect the composite of the thyroid parameters tested. Normal reflects negative TgAA levels, and normal free T4 and TSH concentrations; autoimmune thyroiditis reflects the positive presence of TgAA, reduced free T4 or increased TSH; compensative autoimmune (subclinical) thyroiditis reflects the positive presence of TgAA, normal free T4, and normal or increased TSH; idiopathic hypothyroidism reflects the absence of TgAA, low levels of free T4 and increased TSH; and equivocal reflects an equivocal level of TgAA, normal free T4 and TSH concentrations (Table 1). Equilibrium dialysis testing is the approved OFA diagnostic method used to measure free T4 for all evaluations. Breed, sex, date of birth, and testing date were also recorded. Whether a dog was intact or neutered is not recorded in the OFA database and therefore the sex was simply male or female. Because all dogs had more than one evaluation, initial classifications were compared to the final classification for each dog; final classification was determined based upon the thyroid parameters as defined by OFA classification (see text footnote 1) and required two or more measurements indicating stability of the classification. Dogs with unknown initial classifications were removed from further study. For dogs having only two evaluations, where the second was different than the initial, the final classification was based on the results of the second evaluation. The average ages (in months) of the first and second evaluations were calculated for the entire cohort and per breed, as well as the time (in months) between evaluation 1 and evaluation 2. The OFA ordered categories of thyroid evaluation status, along with the repeated measures on the same animal, required the application of the R-language (23) logistic regression package *ordinal* (24). The ordinal model included terms for sex, breed, a regression on the age (in months) at the time of measurement, a term to distinguish the initial from the final measurement and finally a term for the animal providing the measurement, to accommodate the repeating of ordinal scores. A probit link function, with 4 thresholds to be estimated, facilitated the estimation of unknown parameters, where animal and breed were included as random effects.

**Table 1:** Thyroid parameters underlying the orthopedic foundation for animals classification of thyroid status (16).

Classification	Free T4	Thyroid Stimulating Hormone (TSH)	Thyroglobulin Autoantibodies (TgAA)
Normal	Normal FT4	Normal TSH	TgAA negative
Autoimmune Thyroiditis	Reduced FT4	Increased TSH	TgAA positive
Compensative Autoimmune Thyroiditis (subclinical)	Normal FT4	Normal or increased TSH	TgAA positive
Idiopathic Hypothyroidism	Reduced FT4	Increased TSH	TgAA negative
Equivocal	Normal FT4	Normal TSH	TgAA detectable

## Results

Records from the OFA database yielded 8,309 dogs having multiple thyroid evaluations, representing 161 different pedigree breeds and a small mixed-breed cohort (designated as “hybrid” by OFA), with 4,504 females and 3,805 males (Supplementary Table 1). The number of evaluations ranged from two to eight tests for a given dog with the vast majority having two tests (Supplementary Table 2). Fifty-four dogs had unknown categories for their initial evaluation and were excluded from further consideration, leaving 8,255 dogs. Of these, 8,215 were pedigree dogs and 40 were hybrid dogs, consisting of 4,481 females and 3,774 males (Supplementary Table 1). Males and females were tested with equal frequency at 2.27 and 2.24 average number of evaluations, respectively.

The mean age of the initial thyroid evaluation was 29.7 months (range 2–213 months) and an average of 18.3 ± 16.7 months between the initial and second evaluation (range 1–75 months, Supplementary Table 3). Dogs with an initial to final evaluation of normal (Table 2; Supplementary Table 4, *n* = 3,638) were first tested at an average age of 27.3 months (range 2–148 months). The average age and the time between initial and second evaluation that were recorded as normal for both evaluations are given in Supplementary Table 4 for all individual breeds. Dogs with an initial evaluation of equivocal and a final evaluation of normal (Table 2, *n* = 2,144) were first tested at an average age of 32 months (range 4–213 months). A small percentage of dogs (2.8%, 228 dogs) were tested repeatedly under the age of 12 months with the majority of those 228 (81.6%) being normal for both initial and final evaluations and a very small proportion of those 228 (7.9%) being initially equivocal with a final evaluation of normal. Of the dogs having an initial equivocal evaluation (Table 2), 99.3% were 12 months or older when initially tested and only 22 dogs evaluated under the age of 12 months (range 4–11 months). For these 22 dogs, 1 dog had autoimmune thyroiditis, 2 dogs with compensative autoimmune thyroiditis, 1 dog as equivocal, and 18 were normal in their final evaluations.

**Table 2:** Initial and final classifications in 8,255 dogs evaluated with serial thyroid testing.

Classification	Initial classification total (%)	Final classification total				
		Autoimmune thyroiditis	Compensative autoimmune thyroiditis	Equivocal	Idiopathic hypothyroidism	Normal
Autoimmune thyroiditis	39 (0.5%)	13	15	7	0	4
Compensative autoimmune thyroiditis	996 (12.1%)	36	489	179	7	285
Equivocal	3,140 (38.0%)	7	140	812	37	2,144
Idiopathic hypothyroidism	52 (0.6%)	1	0	18	8	25
Normal	4,028 (48.8%)	7	49	316	18	3,638
		64 (0.8%)	693 (8.4%)	1,322 (16.0%)	70 (0.9%)	6,096 (73.9%)

Upon repeated evaluations, of the 8,255 dogs, 6,096 (73.9%) were recorded as normal. The percentage of dogs in which any classification remained the same from the initial to the final evaluation was 60.1% (4,960/8,255). In initial evaluations, a normal classification was the most frequent (48.8%), followed by equivocal (38.0%), compensative autoimmune thyroiditis (12.1%), idiopathic hypothyroidism (0.6%) and the fewest was inherited autoimmune thyroiditis (0.5%) (Table 2). Final classification following repeated testing revealed that 90.3% of dogs initially classified as normal remained normal, 68.3% of dogs initially classified as equivocal were classified as normal, 28.6% of dogs initially classified as compensative autoimmune thyroiditis were classified as normal, 48.1% of dogs initially classified as idiopathic hypothyroidism were classified as normal, and 10.3% of dogs initially classified as autoimmune thyroiditis were classified as normal (Table 2). The final classification differed from the initial classification in 39.9% (3,295/8,255) of the dogs, with the majority of those being from the equivocal group. Over the entire cohort, 58.2% of the dogs (2,458/4,227) that were initially classified as being something other than normal were then classified as normal after re-evaluation. Conversely, 7.9% (316/4,028) of dogs initially classified as normal received final evaluations of equivocal, 1.2% (49/4,028) were classified as compensative autoimmune thyroiditis at the final evaluation, 0.5% (18/4,028) were re-evaluated as idiopathic hypothyroidism in the final evaluation, and 0.2% (7/4,028) of dogs initially classified as normal received final evaluations of autoimmune thyroiditis. In the final evaluation, 44 dogs initially classified as equivocal (*n* = 37) or compensative autoimmune thyroiditis (*n* = 7) were revised to idiopathic hypothyroidism reflecting an overall revision of 0.8% to idiopathic hypothyroidism in the cohort. The relative proportion of females and males differed significantly (*p* < 0.05) in their final normal and equivocal thyroid classifications (Table 3).

**Table 3:** Final thyroid evaluation classifications for female and male dogs expressed as a percentage.

Sex	Autoimmune thyroiditis	Compensative autoimmune thyroiditis	Equivocal^a^	Idiopathic hypothyroidism	Normal^a^
Female (*n* = 4,481)	0.62	8.77	14.10	0.71	75.79
Male (*n* = 3,774)	0.95	7.95	18.55	1.01	71.54

a Females and males are significantly different from each other *p* < 0.05.

**Figure 1:**
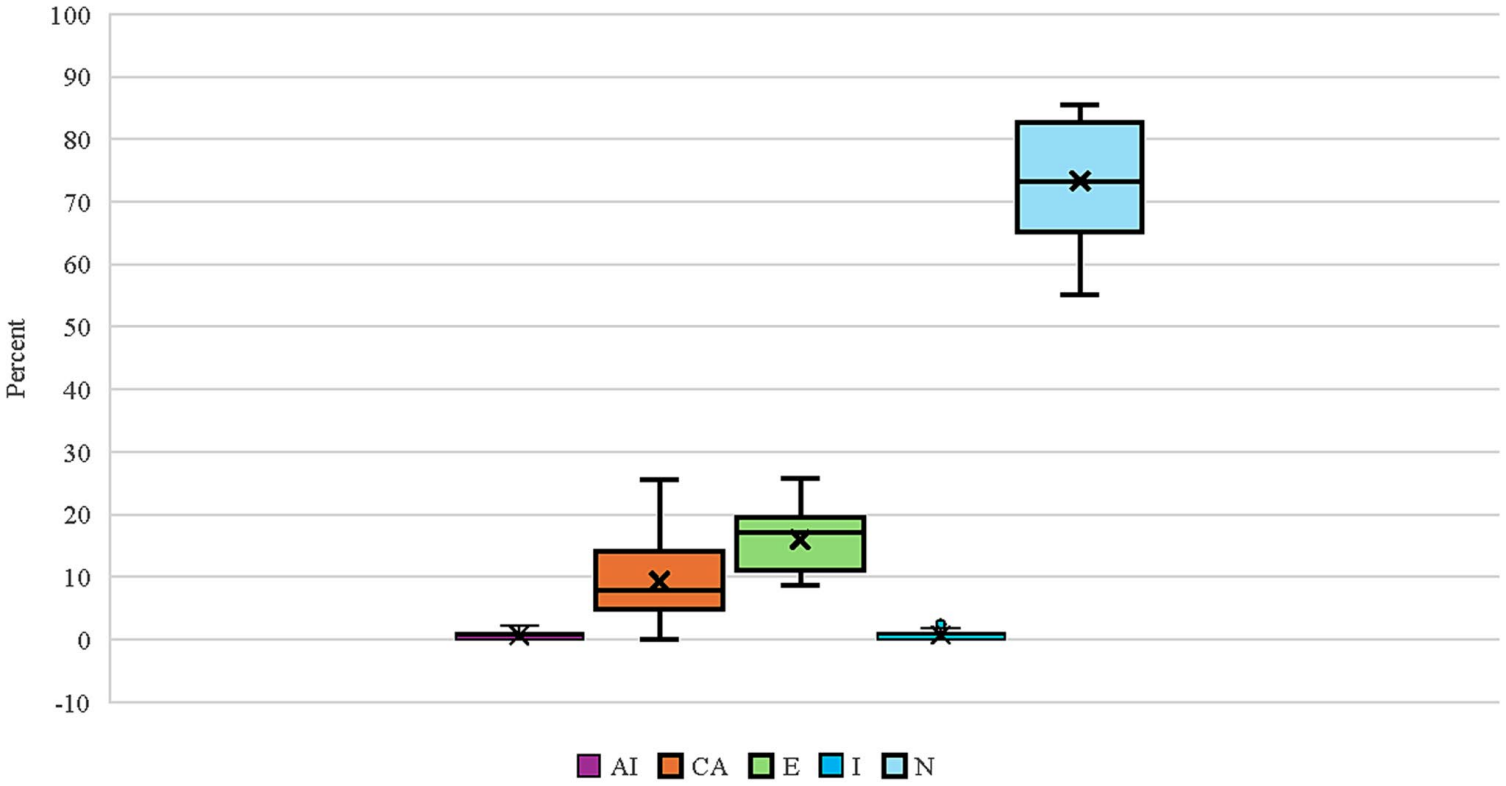
Box and whisker plot for the proportion of final thyroid classifications (AI, Autoimmune thyroiditis; CA, Compensative autoimmune thyroiditis; E, Equivocal; I, Idiopathic hypothyroidism; N, Normal) for 24 breeds having at least 100 individuals evaluated multiple times. The box lines indicate quartiles with the “X” indicating the mean, and the whiskers denoting the minimum and maximum observed evaluations.

Twenty-four breeds had repeated thyroid evaluations on 100 or more individual dogs (Figure 1). Four dog breeds had final evaluations of autoimmune thyroiditis above 1% (Boxers, English Setters, Rhodesian Ridgebacks, and Shetland Sheepdogs) with Rhodesian Ridgebacks significantly different (*p* < 0.05) for autoimmune thyroiditis, compensative autoimmune thyroiditis, and equivocal from the overall cohort proportion (Table 4; raw data in Supplementary Table 5). Of these four breeds, Boxers, English Setters, and Shetland Sheepdogs had less than 60% of dogs evaluated as normal. The Boxer, Doberman Pinscher, German Wirehaired Pointer, Great Dane, and Leonberger all had equivocal evaluations in excess of 20% of the dogs tested. Eight breeds (Bearded Collie, Belgian Tervuren, Bouvier des Flandres, Dalmatian, Giant Schnauzer, Irish Setter, Poodle, and Vizsla) had no dogs evaluated as having autoimmune thyroiditis. Five out of 24 breeds had more than 83% of the dogs evaluated as normal (Australian Labradoodle, Belgian Tervuren, Borzoi, Irish Setter, and Poodle).

**Table 4:** Final thyroid classifications, expressed as a percentage, for breeds having at least 100 individuals evaluated multiple times.

Breed	Number of dogs with multiple evaluations	Autoimmune thyroiditis	Compensative autoimmune thyroiditis	Equivocal	Idiopathic hypothyroidism	Normal
ALL Combined	8,255	0.78	8.39	16.01	0.85	73.85
Alaskan Klee Kai	135	0.74	3.70	12.59	0.00	82.96
Australian Labradoodle	103	0.97	4.85	8.74	0.00	85.44
Basenji	111	0.90	6.31	10.81	0.90	81.08
Bearded Collie	161	0.00	0.00	17.39	0.62	81.99
Belgian Tervuren	194	0.00	3.09	11.86	1.03	84.02
Borzoi	312	0.64	5.45	9.94	0.96	83.01
Bouvier des Flandres	108	0.00	5.56	19.44	1.85	73.15
Boxer	138	2.17	21.01	21.01	0.72	55.07
Dalmatian	274	0.00	16.06	18.98	0.73	64.23
Doberman Pinscher	327	0.61	1.22	22.94	2.45	72.78
English Setter	238	1.68	25.63	16.81	0.00	55.88
German Wirehaired Pointer	138	0.72	15.94	20.29	0.72	62.32
Giant Schnauzer	129	0.00	8.53	19.38	0.00	72.09
Golden Retriever	321	0.93	11.84	12.15	0.31	74.77
Great Dane	640	0.47	7.81	25.78	1.09	64.84
Irish Setter	256	0.00	5.08	10.16	0.39	84.38
Leonberger	131	0.76	9.92	22.14	0.76	66.41
Mastiff	149	0.67	4.70	17.45	0.00	77.18
Nova Scotia Duck Tolling Retriever	114	0.88	14.91	14.04	0.00	70.18
Poodle	433	0.00	4.85	10.62	0.46	84.06
Rhodesian Ridgeback	1,067	1.87	10.31	13.78	0.84	73.20
Shetland Sheepdog	144	2.08	18.75	18.75	0.69	59.72
Vizsla	153	0.00	9.15	10.46	0.65	79.74
Welsh Springer Spaniel	200	1.00	8.00	17.50	2.50	71.00

The average age for an initial evaluation in these 24 breeds was 29.4 months (range 2–157 months), with an average of 18.5 ± 16.7 months between the initial evaluation and the second evaluation (Supplementary Table 6). The Australian Labradoodle had the youngest average age for the initial evaluation at 18 months old (range 3–67 months), whereas the Borzoi had the oldest average age of 35.9 months (range 9–111 months). The Australian Labradoodle, Dalmatian, Doberman Pinscher, Mastiff, and Poodle had dogs evaluated under 6 months of age, the youngest being the Doberman Pinscher and the Poodle, each having one dog evaluated at 2 months of age; in the case of the latter, those dogs were reevaluated over 18 months later and had an initial normal to a final normal evaluation. The Rhodesian Ridgeback, having the most dogs evaluated (Table 4, *n* = 1,067), had an average initial evaluation age of 28.3 months (range 7–157 months) with an average of 19.2 ± 16.2 months between the initial evaluation and the second evaluation (Supplementary Table 6).

## Discussion

This study demonstrated that dogs with normal thyroid evaluations based upon TgAA levels, free T4, and TSH concentrations tend to remain normal over multiple tests, therefore confirming the efficacy of thyroid testing for use in breeding stock selection to reduce the incidence of inherited hypothyroidism. The results also showed that the initial classification of equivocal was most prone to revision with follow-up evaluations. This is not unexpected given that the thyroid parameters detected were unresolved, leading to a classification as an equivocal evaluation. It is known that external factors such as diet (7, 25), exercise (26, 27), or ambient temperature (28) impact measures of thyroid function. Physiological status such as growth phase (29), reproductive state (29), breed, age, size, and medical treatments [reviewed in (30) or females evaluated near estrus (12, 31, 32) also affect thyroid assessments. In this retrospective study, only age, sex, and breed of dogs could be accounted for in the analyses. Age of the dog at the initial classification did not appear to influence the revision with the majority of the dogs (> 97%) which were evaluated initially at 12 months or older. Elevated TgAA levels indicative of subclinical or overt autoimmune thyroiditis are detectable by 12 months of age and precede clinical manifestations of the disease (16).

Because the classification as either equivocal or compensative autoimmune thyroiditis relies solely upon the presence and amount of TgAA with other parameters being normal, those are the classifications that cause the greatest uncertainty with respect to the biological import (21). These two classifications were also observed to have the greatest number of revisions from the initial to the final classification, which is not unexpected because those two classifications are based on a single parameter that may be influenced by many non-thyroid related factors. For example, autoantibodies are found in humans in the absence of disease and their appearance can be transitory in response to environmental factors (33), and it has been proposed in rats (34) and dogs (17, 35) that autoantibody production to thyroglobulin is influenced by sex steroids. It has also been reported that dogs can have circulating antibodies to IgG antibodies (36), and these autologous IgG antibodies can influence the outcome of tests for the presence of other antibodies (36, 37). Thus, other factors beyond true autoimmune thyroiditis may be responsible for the relatively high proportion of initial classifications as equivocal and compensative autoimmune thyroiditis that are then revised with additional testing.

Whether those classifications reflect biological instability of thyroid disease or non-thyroid factors influencing the presence of autoantibodies resulting in erroneous classification was investigated in a prospective study. Egbert et al. (21) found that 42.5% of dogs deemed subclinical (e.g., compensative autoimmune thyroiditis and having positive levels of TgAA, had converted to clinical thyroiditis, whereas 35% remained in the subclinical status having measurable TgAA while being euthyroid; others have shown that more than 50% of subclinical dogs remain subclinical and euthyroid and only 19% converted to clinical hypothyroidism (5). Data from the present study suggest that an initial evaluation based on detectable TgAA levels above a certain threshold [e.g., 10% (38)] could possibly be revised with follow-up evaluations. The high number of equivocal evaluations being revised to normal would support the view that external influences on the presence of detectable TgAA rather than disease variability, underpin those intermediate categories. Additionally, a number of dogs were revised to idiopathic hypothyroidism upon reevaluation, with the majority of those being initially classified as equivocal, indicating the utility of serial testing in accurately defining the thyroid status of a given dog.

Although not every breed was represented in the cohort reported here, similar to the findings of other studies, certain breeds tested demonstrated a predilection to inherited hypothyroidism (2, 39, 40). Those breeds being the Boxer, English Setter, Rhodesian Ridgeback, and Shetland Sheepdog, which presented with significantly higher numbers of autoimmune thyroiditis, compensative autoimmune thyroiditis, and equivocal evaluations in the current study. Additionally, Boxers, English Setters, and Shetland Sheepdogs are known to have other autoimmune conditions (40–42) that are inherited which could contribute to a heightened immune response. This could possibly account for the presence of TgAA in these breeds, leading to the observed elevated numbers of equivocal and compensative autoimmune thyroiditis classifications. Many breeds are suspected to have inherited hypothyroidism in their gene pool, and breed clubs recommend thyroid evaluation as a health check prior to breeding; 49 breeds require thyroid testing (https://ofa.org/chic-programs/ accessed June 16, 2025). Those breeds would be submitted with higher frequency to the OFA database and therefore represent a larger proportion of the study cohort than breeds not considered at risk. Susceptibility to autoimmune thyroiditis in several breeds and the lack of genetic tests available underscore the need for reliable phenotypic assessments. The present study demonstrated that dogs evaluated as normal can be considered to have a very low likelihood of being affected by autoimmune thyroiditis, and that information can assist in reducing the incidence in a given breed. Vigilance in monitoring is still required should a dog exhibit clinical signs of hypothyroidism.

In this study, the data demonstrated that males had a significantly lower proportion of individuals evaluated as normal when compared to females. This was unexpected because female dogs typically have a higher incidence of autoimmune disorders (42) though neutering tends to be associated with a higher frequency of autoimmune conditions (43). Neutered male dogs have been previously shown to have a higher prevalence of hypothyroidism (2). However, in a small study, there were no detected differences in prevalence between neutered males and females (44). (45) found that intact males had the highest risk of hypothyroidism when compared to other sex categories, while it’s been reported that intact females had a significantly lower probability of presenting with hypothyroidism than neutered males and females or intact males (43). In the present study, neuter status was unknown, and whether a dog was intact or neutered could have influenced the observations. The present study had relatively equivalent numbers of males and females in those breeds that were more prone to autoimmune thyroiditis and thus breed sampling bias should not have contributed to the results. A recent study of Eurasian dogs found that males had a greater proportion of TgAA positive hypothyroid diagnoses than females and many fewer males than females in the category of TgAA negative hypothyroid (17). These conflicting published studies, coupled with the present study data, could reflect a more general classification of hypothyroidism rather than specific autoimmune thyroiditis based on the presence of TgAA, or there may have been a greater proportion of neutered dogs in the previously published study cohorts. Clearly, sex is a factor in thyroid assessments and specificity of hypothyroid classification is an important consideration when comparing studies.

A retrospective study is not without limitations. It is possible that once a normal classification is obtained, owners will be less likely to pursue additional testing. However, over 90% of the dogs initially classified as normal remained normal with multiple evaluations and a subset of dogs initially classified as normal (21%) were retested three times and some up to seven times and remained normal in the final classification. Another limitation is the absence of complete medical records for the cohort, therefore, it was not known if a concurrent illness or treatments could have affected the outcome of the results (1). Although the OFA certifications are predominantly used as a health screening test, that does not preclude owners from having administered exogenous thyroid supplementation to dogs initially receiving an equivocal, compensative autoimmune thyroiditis, or autoimmune thyroiditis evaluation. Although if exogenous thyroid medication is given, TSH will be reduced (46) and that will not result in a normal evaluation. Another limitation, noted above, is that neuter status was not recorded and that could also impact the evaluation results. Finally, the transitory nature of autoantibodies in that they decline post-tissue destruction (17) may lead to the conclusion that the absence of detectable TgAA may not be a reliable indicator of normalcy. However, if the thyroid tissue is no longer present, the other thyroid hormonal measures would be disrupted and a normal evaluation precluded. Thus, an evaluation of normal would be reliable.

Despite the limitations of the existing phenotypic testing, assessing thyroid testing by this method when done beyond the age of 12 months, does provide substantive information that can be relied upon for use in breeding decisions to reduce the incidence of the condition (16, 17). Owners that receive an evaluation of hypothyroidism are encouraged to repeat the evaluation at a later date to verify the assessment as has been previously recommended (21). The observation that 50% of those dogs initially evaluated as compensative autoimmune thyroiditis remained classified as compensative autoimmune thyroiditis despite multiple testing suggests that the presence of TgAA while being euthyroid may not be predictive of early stages of autoimmune-mediated hypothyroidism. Whereas elevated levels of TgAA are predictive of autoimmune thyroiditis (16, 17), their presence at low levels is cause for a follow-up assessment. This clearly underscores the need for additional research into immune-mediated hypothyroidism in dogs.

## Data Availability

Publicly available datasets were analyzed in this study. This data can be found at: https://ofa.org/.

## Footnotes

^1^ https://ofa.org/diseases/thyroid/
